# Lrp5 Is Not Required for the Proliferative Response of Osteoblasts to Strain but Regulates Proliferation and Apoptosis in a Cell Autonomous Manner

**DOI:** 10.1371/journal.pone.0035726

**Published:** 2012-05-02

**Authors:** Behzad Javaheri, Andrew Sunters, Gul Zaman, Rosemary F. L. Suswillo, Leanne K. Saxon, Lance E. Lanyon, Joanna S. Price

**Affiliations:** 1 Department of Oral Biology, UMKC School of Dentistry, Kansas City, Missouri, United States of America; 2 Department of Veterinary Basic Sciences, The Royal Veterinary College, Royal College Street, London, United Kingdom; 3 Bristol Veterinary School, Langford House, Langford, Bristol, United Kingdom; Inserm U606 and University Paris Diderot, France

## Abstract

*Although Lrp5* is known to be an important contributor to the mechanisms regulating bone mass, its precise role remains unclear. The aim of this study was to establish whether mutations in *Lrp5* are associated with differences in the growth and/or apoptosis of osteoblast-like cells and their proliferative response to mechanical strain *in vitro.* Primary osteoblast-like cells were derived from cortical bone of adult mice lacking functional *Lrp5* (*Lrp5^−/−^*), those heterozygous for the human G171V High Bone Mass (HBM) mutation (*LRP5* ^G171V^) and their WT littermates (WT*_Lrp5_*, WT_HBM_). Osteoblast proliferation over time was significantly higher in cultures of cells from *LRP5* ^G171V^ mice compared to their WT_HBM_ littermates, and lower in *Lrp5^−/−^* cells. Cells from female *LRP5* ^G171V^ mice grew more rapidly than those from males, whereas cells from female *Lrp5^−/−^* mice grew more slowly than those from males. Apoptosis induced by serum withdrawal was significantly higher in cultures from *Lrp5^−/−^* mice than in those from WT_HBM_ or *LRP5* ^G171V^ mice. Exposure to a single short period of dynamic mechanical strain was associated with a significant increase in cell number but this response was unaffected by genotype which also did not change the ‘threshold’ at which cells responded to strain. In conclusion, the data presented here suggest that *Lrp5* loss and gain of function mutations result in cell-autonomous alterations in osteoblast proliferation and apoptosis but do not alter the proliferative response of osteoblasts to mechanical strain *in vitro*.

## Introduction

Normal bone homeostasis is achieved by balancing the number and activity of bone forming osteoblasts and bone resorbing osteoclasts. In the adult skeleton the mechanical strain engendered within the bone tissue by the activities of normal life acts as a stimulus to regulate this osteoblast/osteoclast balance, thereby controlling bone mass and architecture such that the skeleton is of sufficient strength to withstand the loads placed upon it without damage. There is compelling evidence that the co-ordinated activities of many different signalling pathways function to control osteoblast number and activity both basally and in response to mechanical strain [1]. One such pathway is the Wnt pathway. Engagement of the Wnt ligand with the receptor complex comprising of Frizzled and *Lrp5/6* stimulates activation of the canonical (involving β-catenin activation) and planar cell polarity pathways. Indeed Wnt signalling has been implicated in the regulation of mesenchymal precursor commitment to the osteoblast lineage [2], [3], osteoblast proliferation [4], [5], terminal differentiation [6] and apoptosis [7]–[9]. Humans with an inactivating mutation in the *Lrp5* Wnt co-receptor gene have reduced bone mass [6], [10], [11], whilst individuals with an activating mutation (the G171V mutation) have correspondingly higher than normal bone mass [12]–[14]. Experimental models such as *Lrp5* knockout mice [4] or mice expressing human *LRP5* transgene containing the G171V activating mutation (*LRP5* ^G171V^) [7] generally recapitulate the situation in humans and have low and high bone mass respectively.

Sawakami *et al*., (2006) provided *in vivo* evidence that the Wnt pathway may play a role in mediating bone's adaptive response to loading, by demonstrating that mice lacking functional *LRP5* have an impaired cortical bone response to ulna loading [15]. In a recent study that analysed multiple bone responses to graded strains we also demonstrated that absence of *Lrp5* activity due to the *Lrp5^−/−^* mutation reduces the osteogenic effects of loading in male (but not female) mice, whilst the presence of the *LRP5* ^G171V^ mutated gene was associated with increased mechano-responsiveness [16]. However, this study supported only a limited gender-related role for LRP5 function in mediating bone's adaptive response to mechanical loading *in vivo*.

Given that *Lrp5* status *in vivo* impacts basal and mechanically influenced bone mass, we sought to investigate *in vitro* whether primary long-bone-derived osteoblast like cells derived from both the *Lrp5^−/−^* or *LRP5* ^G171V^ mice displayed cell autonomous differences in their basal growth and apoptosis rates that could explain these physiological phenotypes. We also measured proliferation in these cells following exposure to mechanical strain in order to determine whether *Lrp5* functionality regulated the magnitude of the strain-related response and/or altered the ‘threshold’ at which a proliferative response to strain was engendered.

## Materials and Methods

### Ethics statement

The genetic background of *LRP5* ^G171V^ and *Lrp5* ^−/−^ is the mouse strain C57BL/6 (Charles River Laboratories, Margate, Kent, U.K). *LRP5* ^G171V^ transgenic and *Lrp5^−/−^* mice were a gift from Babij *et* *al*., (2003) and Kato *et* *al*., (2002) respectively [4], [7]. Mice from both colonies were housed up to 5 per cage in polypropylene cages with wood chip and paper bedding and provided standard mouse chow and water ad libitum. Weaners up to 8 weeks of age were fed a standard rodent breeding diet and thereafter a standard rodent maintenance diet (Special Diet Services, South Witham, UK). All of the procedures conducted in the facility were carried out in accordance with the UK Animals Act (Scientific Procedures) 1986 under a UK Government Home Office project license number PIL70/6350, reviewed and approved by the Royal Veterinary College Local Ethical Review Committee (London, UK).

### Cell extraction

At 19 weeks of age, *LRP5* ^G171V^ and *Lrp5* ^−/−^ mice and their WT littermates (WT_HBM_ and WT*_Lrp5_*) were euthanized by means of cervical dislocation. Primary osteoblast-like cells were isolated from femur, radii, ulnae and humerus as described previously [17]. The osteoblast-like cells were maintained in Dulbecco's minimal essential medium (DMEM) without phenol-red, 2 mM L-glutamine (Life Technologies Ltd.), 100 U/ml penicillin (Life Technologies Ltd.), and 100 μg/ml streptomycin (Invitrogen Ltd, Paisley, Scotland, UK (Life Technologies Ltd.) supplemented with 10% heat-inactivated foetal calf serum (FCS) (LabTech International, East Sussex, UK) and were incubated at 37°C in a humidified 5% CO2 incubator. Only first passage cells (P1) were used.

### Cell proliferation rates

Primary osteoblast-like cells isolated from female and male LRP5^G171V^ and Lrp5^−/−^ mice and their WT littermates and experiments were repeated three times (2 mice of each gender and genotype were used). The cells were seeded evenly onto custom-made sterile, tissue culture-treated plastic strips (66 mm×22 mm) (Nunc, Dossel, Germany) at a density of 100,000 cells/strip. Four strips were incubated together in quadriPERM 4-well plates (Greiner Bio-One, Stonehouse, UK) in DMEM supplemented with 10% FCS. Cells were fixed for 10 minutes in absolute ice-cold MeOH (VWR International) 2, 4, 6 and 8 days after seeding followed by one wash with PBS. To quantify cell number, cells were stained with propidium iodide (INCYTO, Seoul, South Korea) and counted using a Microchip Type Automatic Cell Counter according to the manufacturer's instructions (INCYTO, Seoul, South Korea).

### TUNEL staining

For each experiment and each experimental group, primary osteoblast-like cells were extracted from the femur, humerus, radius and ulna of one 19 week old mouse. These studies were repeated 3 times and thus included 3 mice per group. 20,000 first passage primary osteoblast-like cells derived from female and male *Lrp5* ^−/−^, *LRP5* ^G171V^ and WT_HBM_ mice were seeded onto 24-multiwell plates (16 mm in diameter) and cultured in DMEM supplemented with 10% FCS. Six wells per treatment group were used. After 72 hours the cells were washed twice with serum free DMEM and thereafter were incubated with fresh DMEM containing 0.1%, 2.5% or 10% FCS. After 48 hours the cells were fixed with 4% paraformaldehyde. Nuclear DNA fragmentation as a measure of apoptosis was evaluated by the terminal deoxynucleotidyl transferase-mediated dUTP nick-end labelling (TUNEL) method using DeadEnd Fluorometric TUNEL System (Promega, Madison, WI) according to the manufacturer's protocol. Vectashield mounting medium with DAPI (Vector Laboratories) was used for the counterstaining of nuclei. Nuclear TUNEL positive cells were judged to be apoptotic. Total cell number and the percentage of TUNEL positive cells were determined in 5 microscopic fields per well at 10× magnification. The fields were photographed using a Leica Q550IW fluorescent microscope with a DC 500 Leica digital camera and cells were counted from the computer screen with the assistance of the Leica Qwin computer programme (Leica, Solms, Germany).

### Cell proliferation in response to mechanical strain

Primary osteoblast-like cells isolated from female and male LRP5^G171V^ and *Lrp5* ^−/−^ mice and their WT littermates were seeded onto custom-made sterile, tissue culture-treated plastic strips (Nunc, Dossel, Germany) at a density of 100,000 cells/strip (3 mice of each gender and genotype were used and experiments were repeated three times). Four strips were used per condition and were incubated together in quadriPERM 4-well plates (Greiner Bio-One, Stonehouse, UK) maintained in DMEM containing 10% FCS for 5 days. Mechanical strain was applied using a loading jig that applies four-point bending to each strip with minimal fluid perturbation as described previously [17]. Cells were subjected to a single period of 600 cycles of four-point-bending at a frequency of 1 Hz. This generated a peak strain of 2500, 2900 or 3400 με. Following strain treatment the strips and media were replaced into the 4-well plates and incubated for 48 hours. Control strips were placed in wells of the loading apparatus and subjected to similar perturbation of surrounding media as experienced by the strained strips. However, these strips were not subjected to bending and thus the cells that were plated onto them were not subjected to strain. Cells were stained with propidium iodide (INCYTO, Seoul, South Korea) and counted using a Microchip Type Automatic Cell Counter according to the manufacturer's instructions (INCYTO, Seoul, South Korea).

### Statistical Analysis

Statistical significance was determined by a 2-tailed unpaired Student's t-test or one way analysis of variance (ANOVA) followed by the post hoc (Bonferroni) multiple comparisons between treatment groups using SPSS statistics package version 16 for Windows (SPSS, Chicago, IL, USA). The mixed model analysis was performed using SAS system v9.0 (SAS Institute, Cary, NC, USA). Cell doubling time were calculated using GraphPad Prism v5.0 software for Windows (GraphPad Software Inc., San Diego, CA) by nonlinear regression (exponential growth equation) analysis. Least significant difference was determined and p<0.05 considered statistically significant. Data displayed as mean ± SEM.

## Results

### Osteoblast proliferation over time *in vitro*

**Figure 1:**
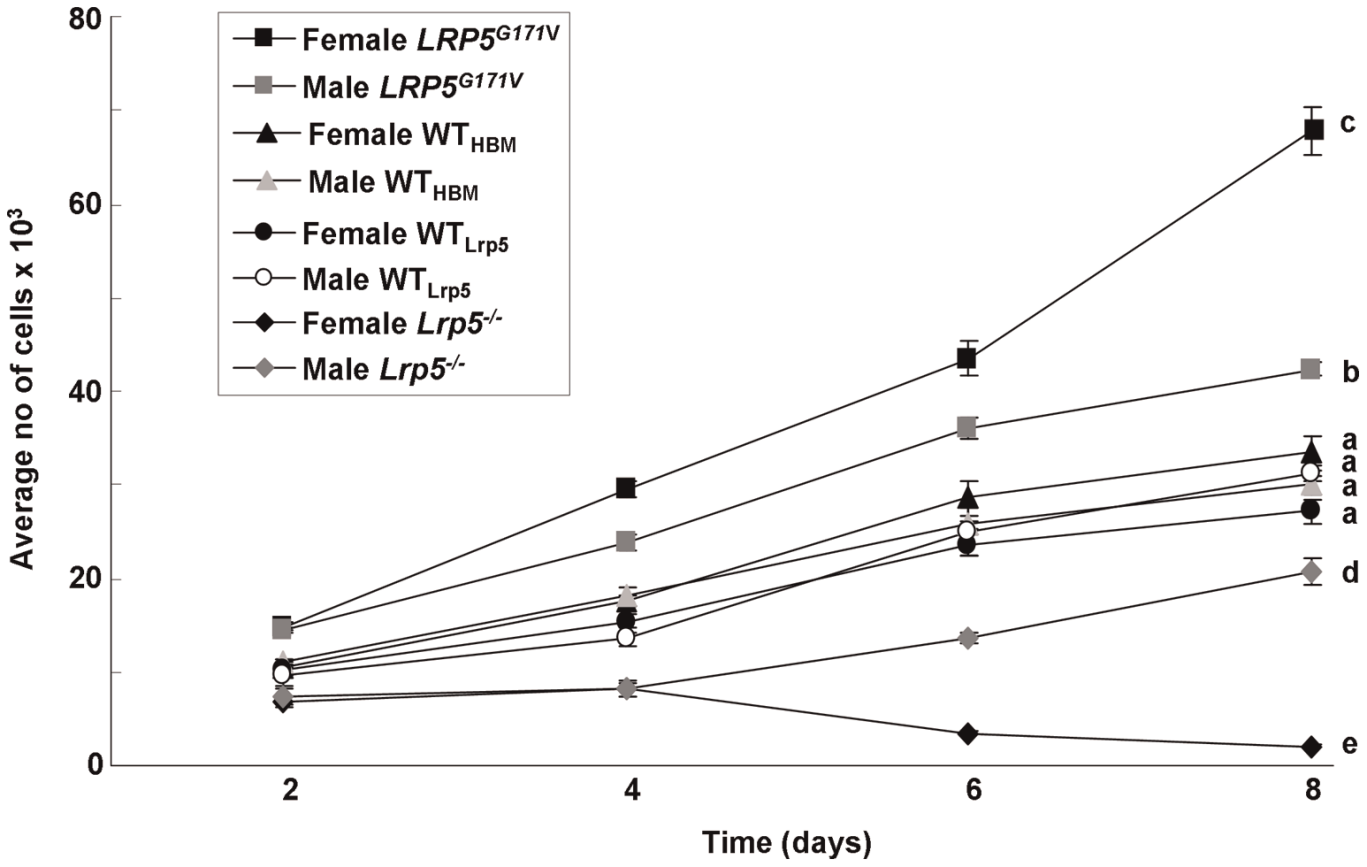
Proliferation of primary osteoblast-like cells derived from female and male *LRP5* ^G171V^ and *Lrp5* ^−/−^ mice and their WT littermates. Osteoblast-like cells were cultured over 8 days and were fixed in absolute ice-cold MeOH on day 2, 4, 6 and 8. Cell's nuclei were stained using propidium iodide and counted using Microchip Type Automatic Cell Counter machine. Results are the mean ± SEM of three independent experiments. N = 4. Groups with the same letter are not significantly different. b vs. a  =  P<0.001. a + b vs. c  =  P<0.001.

Primary osteoblast-like cells extracted from the long bones of female and male *LRP5* ^G171V^ and *Lrp5* ^−/−^ mice and their WT littermates were cultured for 2, 4, 6 or 8 days (Fig. 1). There was a main effect of genotype (p<0.001) and time in culture on cell number (p<0.001). The post-hoc analysis showed that cells from *LRP5* ^G171V^ mice proliferated faster than cells from the WT_HBM_ littermates (p<0.001), whereas cells from *Lrp5* ^−/−^ mice proliferated more slowly than cells from WT*_Lrp5_* littermates. A gender and genotype interaction was detected (p<0.001) together with interaction between gender, genotype and time (p<0.001). The post-hoc analysis also showed that cells from female *LRP5* ^G171V^ mice grew faster than cells from male *LRP5* ^G171V^, male and female WT_HBM_ and male and female *Lrp5* ^−/−^ mice (p<0.001). Furthermore, female *Lrp5* ^−/−^ cells proliferated more slowly compared to male *Lrp5* ^−/−^ cells (p<0.001). Doubling time calculated from the regression analysis of each genotype is shown in table 1.

**Table 1:** Cell population doubling time of primary osteoblast-like cells derived from female and male *Lrp5* **^−/−^**, *LRP5* **^G171V^** and their WT littermates.

Genotype	Double time (days)
Female WT_HBM_	3.42
Male WT_HBM_	3.26
Female WT_Lrp5_	3.26
Male WT_Lrp5_	3.36
Female *LRP5* ^G171V^	2.52
Male *LRP5* ^G171V^	2.96
Female *Lrp5* ^−/−^	−4.34
Male *Lrp5* ^−/−^	4.14

Doubling time in days between 2 and 8 days of culture of primary osteoblast-like cells isolated from female and male *Lrp5* ^−/−^, *LRP5* ^G171V^ and their WT littermates. Cell doubling time were calculated using GraphPad Prism v5.0 software for Windows (GraphPad Software Inc., San Diego, CA) by nonlinear regression (exponential growth equation) analysis.

### TUNEL staining of cells exposed to serum depletion

**Figure 2:**
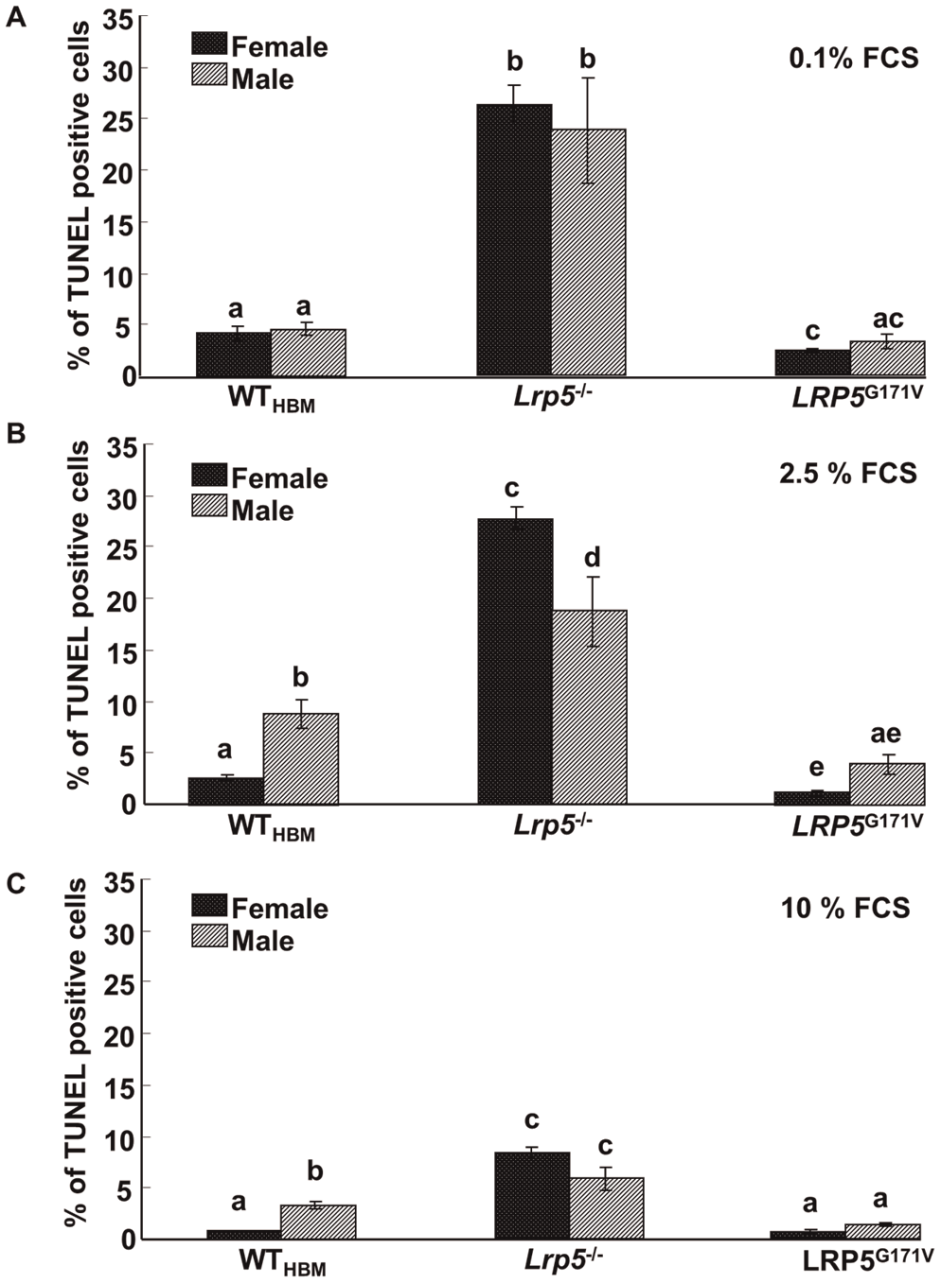
Percentage of apoptosis in osteoblast-like cells 48 hours after treatment with 0.1% (A), 2.5% (B) and 10% (C) serum. The TUNEL staining was used to determine the percentage of apoptotic cells in primary osteoblast-like cells derived from male and female WT_HBM_, *Lrp5* ^−/−^ and *LRP5* ^G171V^ mice. Results are mean ± SEM of three independent experiments. Groups with the same letter are not significantly different.

Having observed that cells from different genotypes of mice proliferated at different rates, we sought to establish whether this reflected a difference in the rate of apoptosis. Significantly higher levels of TUNEL stained cells were observed in cultures from *Lrp5* ^−/−^ mice compared to cultures from WT_HBM_ and *LRP5* ^G171V^ mice (p<0.001) (Fig. 2). In contrast, the percentage of TUNEL stained cells from *LRP5* ^G171V^ mice was significantly lower compared to *Lrp5* ^−/−^ and WT_HBM_ cells (p<0.001). There was an effect of gender, genotype and serum concentration on percentage of TUNEL stained cells (p<0.001) and an interaction between genotype and serum concentration was also detected (p<0.001). In 0.1 and 2.5% serum the percentage of TUNEL positive osteoblast-like cells was significantly higher in cultures of female and male *Lrp5* ^−/−^ mice compared to cultures of female and male WT_HBM_ and *LRP5* ^G171V^ cells (p<0.001 and p<0.01 respectively). Similar to the situation in 0.1 and 2.5% serum, the percentage of TUNEL positive cells in 10% serum was significantly higher in cultures of female and male *Lrp5* ^−/−^ cells compared to female and male WT_HBM_ and *LRP5* ^G171V^ cells (p<0.05). Gender related differences in the percentage of TUNEL positive cells were also observed within each genotype; in 2.5% and 10% serum the percentage of TUNEL positive cells was higher in cultures of male WT_HBM_ cells compared to cultures of female WT_HBM_ cells (p<0.05) and no gender differences in TUNEL positive cells was observed in 0.1% serum. In contrast, in 2.5% serum the percentage of TUNEL positive cells was higher in cultures of female *Lrp5* ^−/−^ cells compared to cultures of male *Lrp5* ^−/−^ cells (p<0.01) and no gender differences in 0.1% and 10% was observed.

### Osteoblast proliferation in response to mechanical strain *in vitro*

**Figure 3:**
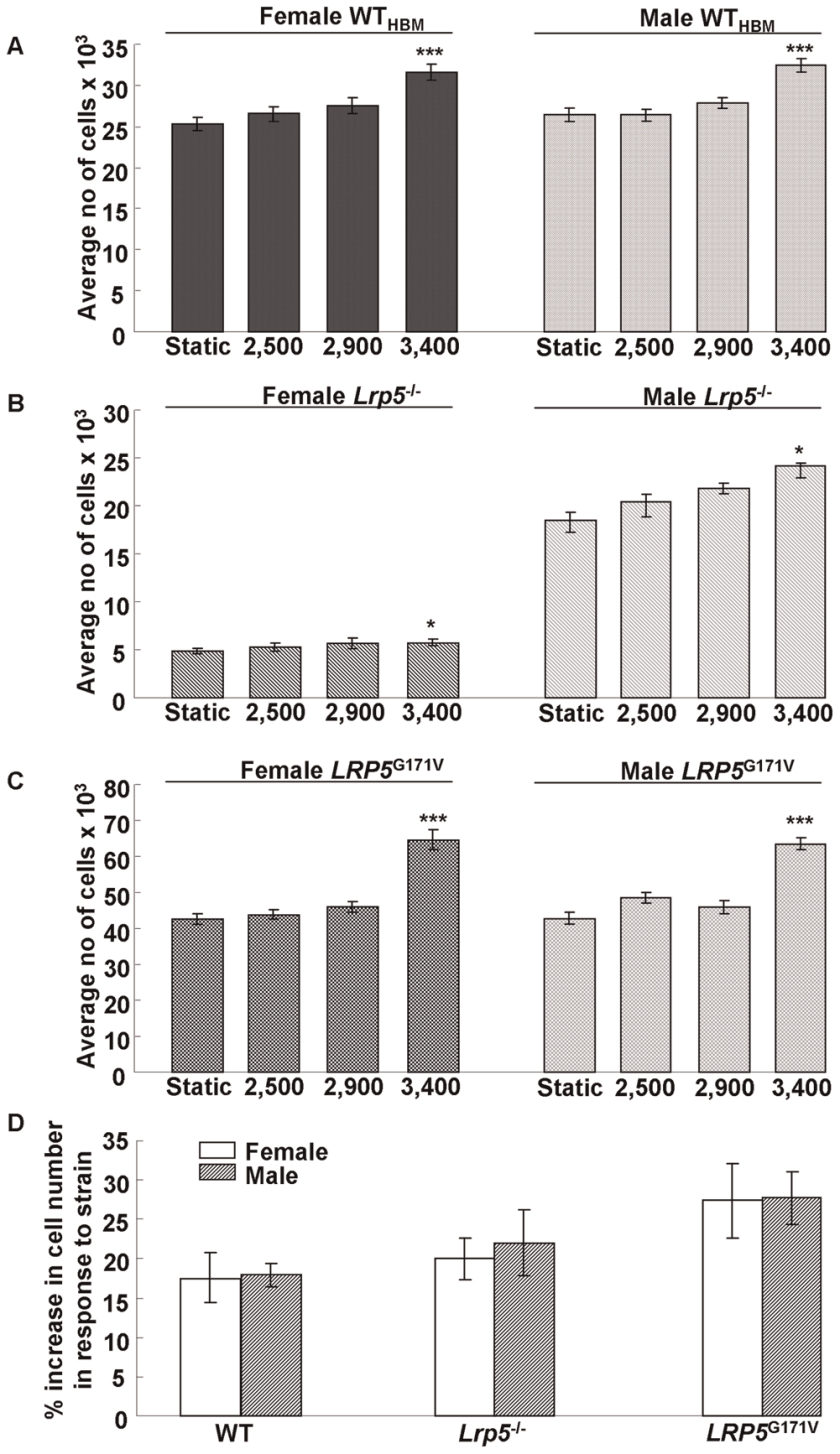
Effects of 2500, 2900 and 3400 με on proliferation of primary osteoblast-like cells derived from female and male WT (A), *Lrp5* ^−/−^ (B) and *LRP5* ^G171V^ (C) mice. Changes in absolute number of cells between static and strain of both genotypes and genders are shown. Results are mean ± SEM of three independent experiments. Experiments were repeated 3 times. No significant differences at 2500 and 2900 με were observed. ***p<0.001 and *p<0.05 compared with the static control within the gender. (D) The effects of 3400 με on proliferation of primary osteoblast-like cells derived from female and male *LRP5* ^G171V^ and *Lrp5* ^−/−^ mice and their WT littermates. Percentage differences between static and strain of both genotypes and genders are shown. Results are the mean ± SEM of three independent experiments. Experiments were repeated 3 times. There were no significant differences between groups.

The proliferative response of osteoblast-like cells cultured from each gender and genotype of mice to different magnitudes of mechanical strain is shown in figure 3. All groups showed no response at 2,500 με and 2,900 με however, for each gender and genotype (p<0.001) a significant increase in cell number was detected at 3,400 με (Figs. 3A, B and C). We also calculated the percentage increase in cell number between the control (static) and strained groups and found no significant differences between female and male or *LRP5* ^G171V^, *Lrp5* ^−/−^ and WT cells (Fig. 3D).

Mixed model analysis showed a main effect of gender (p<0.001), genotype (p<0.001) and strain (p<0.001) on cell number. A genotype and strain interaction was detected (p<0.006) and an interaction between gender, genotype and strain (p<0.001). As described previously, the number of cells in static control cultures of female and male *LRP5* ^G171V^ cells was significantly higher than in *Lrp5* ^−/−^ and WT_HBM_ cultures. This reflects significant differences in basal proliferation between *LRP5* ^G171V^, *Lrp5* ^−/−^ and WT cells (Fig. 1).

## Discussion

### *LRP5* plays a role in osteoblast proliferation

The key finding from this study was that absence of *Lrp5* function in osteoblastic cells derived from the cortical bone of adult mice was associated with decreased proliferation as evidenced by an increase in cell population doubling time compared with WT*_Lrp5_* cells *in vitro*. Conversely, presence of the *LRP5* ^G171V^ mutation in osteoblastic cells was associated with increased proliferation compared with *Lrp5* ^−/−^ and WT cells. Cell population doubling times were comparable in the cultures of female and male WT_HBM_ and WT*_Lrp5_* cells, suggesting that WT cells from both backgrounds have a similar proliferation rate. These findings provide evidence that the presence of functional *LRP5* protein plays an important role in the regulation of osteoblast proliferation, which is in broad agreement with previous studies [4], [6].

These results are not consistent with those of Yadav *et* *al*., (2009) who observed no significant differences between the proliferation of *Lrp5* ^−/−^ and WT*_Lrp5_* osteoblast-like cells [18]. However, differences between our two studies could potentially explain these apparently contrasting observations. The first relates to the site of origin of the cells; the study described here used osteoblast-like cells derived from long bones, whereas Yadav *et al*, (2009) used calvarial derived osteoblast-like cells. Several years ago our group demonstrated that osteoblastic cells from long bones and calvariae respond differently to a strain-related stimulus [19]. More recently another study confirmed that primary osteoblast-like cells derived from calvariae or long bones are both phenotypically different in vitro and also are significantly different at the level of gene expression [20]. Second, the age of animals was very different in the two studies; Yadav *et al* (2009) derived osteoblast-like cells from new born mice whereas cells from adult (19 week old) mice were used in the present study. Because pre-osteoblasts and osteoblasts from young humans and animals proliferate more rapidly than cells from older animals [21]–[29], this potentially could ‘mask’ the effect on proliferation of the loss of *Lrp5.* It must also be considered that the long-term loss of *Lrp5* (from birth to 19 weeks of age) has different effects to loss of the gene during development only. Kato *et al.,* (2002) demonstrated that bone formation was normal in *Lrp5^−/−^* mice at 17.5 days post coitum and at birth there was only a subtle delay in osteogenesis. However, the ossification defect in these mice became more pronounced with age and reflected a defect in osteoblast proliferation. Finally, different promoters were used in the two studies. The *LRP5* ^G171V^ mice used for the present study were generated by Babij *et* *al*. (2003) using the 3.6 kb fragment of the collagen type 1 promoter to drive the expression of the transgene in pre-osteoblasts and osteoblasts, with minimal to no expression in other cell types [30], [31]. In contrast, Yadav *et* *al*., (2009) used the 2.3 kb collagen type I promoter –cre to replace one copy of the endogenous murine *Lrp5* gene with one copy of *Lrp5* carrying the high bone mass G171V mutation. This generated mice with one WT and one G171V allele, rather than two WT and one G171V allele as used here. The 2.3 kb promoter driving *Cre* expression is activated later in the osteoblast differentiation pathway than the 3.6 kb promoter, and is thus active in more mature osteoblasts [30].

We have also found that the effect of *Lrp5* mutations on osteoblast proliferation was more pronounced in cells from female mice, such that *Lrp5* gain of function stimulated whilst *Lrp5* loss of function impaired proliferation. We have previously shown that ERα is required for β-catenin function in response to strain and it is now apparent that this is mediated, at least in part, by the non-genomic signalling effects of ER involved with IGFIR's action [32], [33]. This suggests that there are gender differences in LRP5-β-catenin signalling in osteoblasts, with females being more affected by changes in its activity because of potential interactions with the E2/ERα IGF-I signalling pathways. Interestingly, it has been shown that bone marrow stromal cells isolated from young women express higher levels of the ER and the ERR target gene Wnt11. Conversely, male BMSCs express higher levels of Wnt 16, which has two isoforms associated with either senescence or proliferation [34], [35].

### *LRP5* is involved in osteoblast apoptosis

Having demonstrated differences in proliferation of cells from different genotypes, we studied the effect of *LRP5* mutations on TUNEL staining which showed that loss of *LRP5* function increased apoptosis in primary osteoblast-like cells from *Lrp5* ^−/−^ mice. This result suggest that the low bone mass phenotype observed *in vivo* may reflect, at least in part, high levels of apoptosis in the bone cells of these mice. This finding is in agreement with a previous *in vitro* study in which apoptosis in calvarial-derived osteoblast-like cells from *Lrp5* ^−/−^ mice was shown to be higher compared to WT cells [9]. However, our data is not in agreement with a previous *in vivo* study by Kato *et al*., (2002), in which no difference in osteoblast apoptosis rates were observed in calvarial sections from *Lrp5* ^−/−^ mice. The *LRP5* G171V mutation seems to provide some protection against apoptosis induced by serum depletion however, the effect of this mutation on osteoblast apoptosis does not appear to be as significant as the loss of *LRP5* function. Notwithstanding, our findings support the idea that *LRP5* is a critical component in the regulation of bone cell apoptosis [7]–[9], [36], [37].

### LRP5 is not required for the proliferative response of osteoblasts to strain

One of the effects of mechanical strain is to stimulate proliferation of cells that are, or will become, osteoblasts [38]–[47]. In the studies reported here neither absence of *LRP5* function nor the presence of the *LRP5* ^G171V^ mutation altered the proliferative response of cortical derived primary osteoblast-like cells to mechanical strain *in vitro*. These data are in agreement with two recent *in vitro* studies which suggested that the strain-induced activation of β-catenin does not require *LRP5* [33], [48], [49], although a previous *in vivo* study had shown that the loading response was abolished in mice lacking *LRP5* [15].

Neither did the *Lrp5* ^−/−^ or the *LRP5* ^G171V^ mutation alter the ‘minimum effective strain’ at which strain engendered a proliferative response, thus our findings are not consistent with *in vivo* evidence that a lower strain threshold is sufficient to induce cortical bone formation in *LRP5* ^G171V^ mice [50], [51]. We have also recently demonstrated that the increased load-induced osteogenesis in the cortical and cancellous bone of mice with the *LRP5* ^G171V^ mutation is more pronounced in females than males [16]. One possible explanation for the apparent differences between our *in vitro* data and *in vivo* findings is that *LRP5* mutations may alter the strain responsiveness in osteocytes rather than osteoblasts. For example, differences in responses to fluid flow shear stress have been reported in osteocytes versus osteoblasts [49], [52], [53]. Our *in vitro* model includes osteoblasts with little or no osteocytic component and so does not replicate the complex context of cortical bone.

In conclusion, we have provided data to demonstrate that in cortical bone-derived primary osteoblast-like cells from adult mice, *LRP5* is an integral component of the signalling pathways that regulate cell proliferation and apoptosis. However, it is not required for the proliferative response of these cells to mechanical strain. The intrinsically higher rate of proliferation and reduced apoptosis observed in the *LRP5* ^G171V^ osteoblastic cells may result in an increased amount of new bone being formed which would partially explain the high bone mass seen in individuals with this mutation.
